# Impact of Outdoor Air Pollutants Exposure on the Severity and Outcomes of Community-Acquired Pneumonia in Gabes Region, Tunisia

**DOI:** 10.7759/cureus.66578

**Published:** 2024-08-10

**Authors:** Hamida Kwas, Harish Rangareddy, Hayfa H Rajhi

**Affiliations:** 1 Pulmonology, University Hospital of Gabès, Gabès, TUN; 2 Biochemistry, Haveri Institute of Medical Sciences, Haveri, IND; 3 Analysis Laboratory Research, University Hospital of Gabès, Gabès, TUN

**Keywords:** community acquired pneumonia., ozone, humidity, sulfur dioxide, particulate matter

## Abstract

Background

Acute community-acquired pneumonia (CAP) is considered the leading cause of infectious death worldwide. Air pollution and prolonged exposure to airborne contaminants have been implicated in various respiratory conditions, including asthma and chronic obstructive pulmonary disease (COPD). However, the specific impact of air pollution on pneumonia, particularly CAP, remains underexplored. Given the rising levels of urban air pollution and its potential health ramifications, our study aimed to examine the association between exposure to outdoor air pollution and severity as well as the outcomes of pneumonia cases requiring hospitalization.

Methodology

A cohort analytical study with retrospective data collection was carried out in the pulmonology department of the Gabès University Hospital between January and October 2022. We compared levels of particulate matter less than or equal to 10µm in aerodynamic diameter (PM_10_), sulfur dioxide (SO_2_), ozone (O_3_), moisture and ambient temperature with severity and outcomes of pneumonia requiring hospitalization. The choice of these specific pollutants and environmental factors was based on their established impact on respiratory health and their prevalence in the study region.

Results

Increased sulfur dioxide (SO2) levels were associated with increased use of non-invasive ventilation (NIV) (r = 0.400). Higher levels of particulate matter (PM10) were significantly associated with the development of lung abscesses. Similarly, increased humidity and ambient temperature were strongly correlated with the development of lung abscesses. Increased air SO2 levels were correlated with a higher CURB65 score (r = 0.299). High outdoor SO2 levels and increasing moisture content were associated with increased Pneumonia Severity Index (PSI) score (r = 0.303 and = 0.310, respectively). Higher levels of PM10 were associated with an increased risk of pleural effusion, a serious complication of pneumonia. Finally, higher ambient temperatures were correlated with more extensive opacities on chest X-rays (r = 0.706), suggesting the severity of pneumonia.

Conclusion

This study highlights the significant associations between environmental factors and various clinical parameters in pneumonia patients. The findings underscore the importance of considering environmental exposures, such as air quality and weather conditions, in understanding and managing the severity of pneumonia.

## Introduction

Owing to the increasing industrial and urban activities, the air is becoming increasingly laden with pollutants. These air pollutants pose a significant health risk to the community, causing many respiratory diseases as well as the risk of death [1]. Atmospheric pollution still poses a major health threat worldwide. Acute community-acquired pneumonia (CAP) is considered the leading cause of infectious death worldwide. It has been reported that over 90% of the world’s population lives in areas where the air pollution level exceeds the World Health Organization (WHO) guideline limits [2-4]. While pneumonia is predominantly bacterial in origin, various external factors exacerbate its development and influence its prognosis [5].

The particulate matter (PM) is a mixture of contaminated components resulting from various pollutant emission sources. These fine particles can reach the alveoli, where they are phagocytized by macrophages and neutrophils, releasing inflammatory mediators that cause throat irritation. Additionally, these particles have been linked to an increased risk of pneumonia and myocardial infarction, depending on air pollutant levels [6, 7]. According to the World Health Organization (WHO), the mean limit of acceptability of particulate matter (PM2.5) is 25 μg/m^3^ per day [5, 8]. Seasonal and environment’s climatic parameter variations closely affect the particulate matter levels [9]. Studies have hypothesized that short-term exposure to certain ambient air pollutants including particulate matter with an aerodynamic diameter ≤2.5 μm (PM2.5), particulate matter with an aerodynamic diameter ≤10 μm (PM10), sulfur dioxide (SO2), nitrogen dioxide (NO2), carbon monoxide (CO), and ozone (O3), would be associated with increased risk of hospital admission for pneumonia [10-14].

Air pollution and prolonged exposure to airborne pollutants are linked to different respiratory issues. Research has shown that air pollution is linked to lung cancer, asthma, pneumonia, and exacerbating respiratory allergies [1]. Nevertheless, only a small number of research projects have directly addressed the consequences of air pollution on pneumonia. This research gap led us to study how outdoor air pollution affects pneumonia. The objective of this study was to explore the association between exposure to outdoor air pollution and the severity and outcomes of pneumonia cases requiring hospitalization.

## Materials and methods

A cohort analytical study with retrospective data collection was conducted in the Pulmonology Department of Gabès University Hospital from January 2022 to October 2022. The study focused on patients hospitalized for community-acquired pneumonia (CAP). The inclusion criteria for diagnosing CAP were based on:

Clinical Criteria: Signs suggestive of a lower respiratory infection, including cough, purulent sputum, chest discomfort, recent onset dyspnea, and focal auscultatory signs.

Radiological Criteria: Presence of a new radiological abnormality not previously known, such as alveolar infiltrate with air bronchogram. Initial imaging workup included chest X-rays to identify infiltrates or effusions, enhancing diagnostic accuracy. Computed tomography (CT) scans were also utilized for more detailed imaging, such as identifying lung abscesses and bilateral pneumonia.

Laboratory Criteria: Blood work included a complete blood count with differentials and serum electrolytes with renal function tests to confirm evidence of inflammation and assess severity. The laboratory criteria considered for CAP included neutrophilic leukocytosis and high C-reactive protein (CRP) level (>10mg/L).

Patients were excluded from the study if they met any of the following criteria:

1. Age: Patients aged 14 years or younger.

2. Lack of Diagnostic Criteria: Absence of the clinical, radiological, and laboratory criteria for CAP.

3. Nosocomial Pneumonia: Suspected nosocomial pneumonia, defined as pneumonia appearing more than 48 hours after hospitalization or within 14 days of a previous hospitalization.

4. Immunosuppression: Presence of constitutional or acquired immunosuppression associated with a risk of opportunistic infections, such as HIV infection, progressive neoplasia, immunosuppressive treatment, or long-term corticosteroid therapy.

Severity assessment tools such as CURB-65 (confusion, urea greater than or equal to 20 mg/dL, respiratory rate greater than or equal to 30/min, blood pressure systolic less than 90 mm Hg or diastolic less than 60 mm Hg) and the Pneumonia Severity Index (PSI) were used to assist in determining the treatment setting, such as outpatient versus inpatient care. While these tools provided valuable insights, their accuracy was limited when used alone without practical clinical judgment.

Data collection

Clinical Data Collection

Patient demographics (age and gender), history, symptoms and clinical signs observed during hospitalization were among the clinical data that was gathered. Laboratory data included measurements of white blood cells, hemoglobin, C-reactive protein, and creatinine. Radiological information centred on parenchymal injury detected by chest X-rays or CT scans. The progression of the clinical condition was evaluated at three different intervals: shortly after hospital admission (48-72 hours), prior to hospital discharge, and 15 days after discharge.

Metrological Data Collection

**Figure 1:**
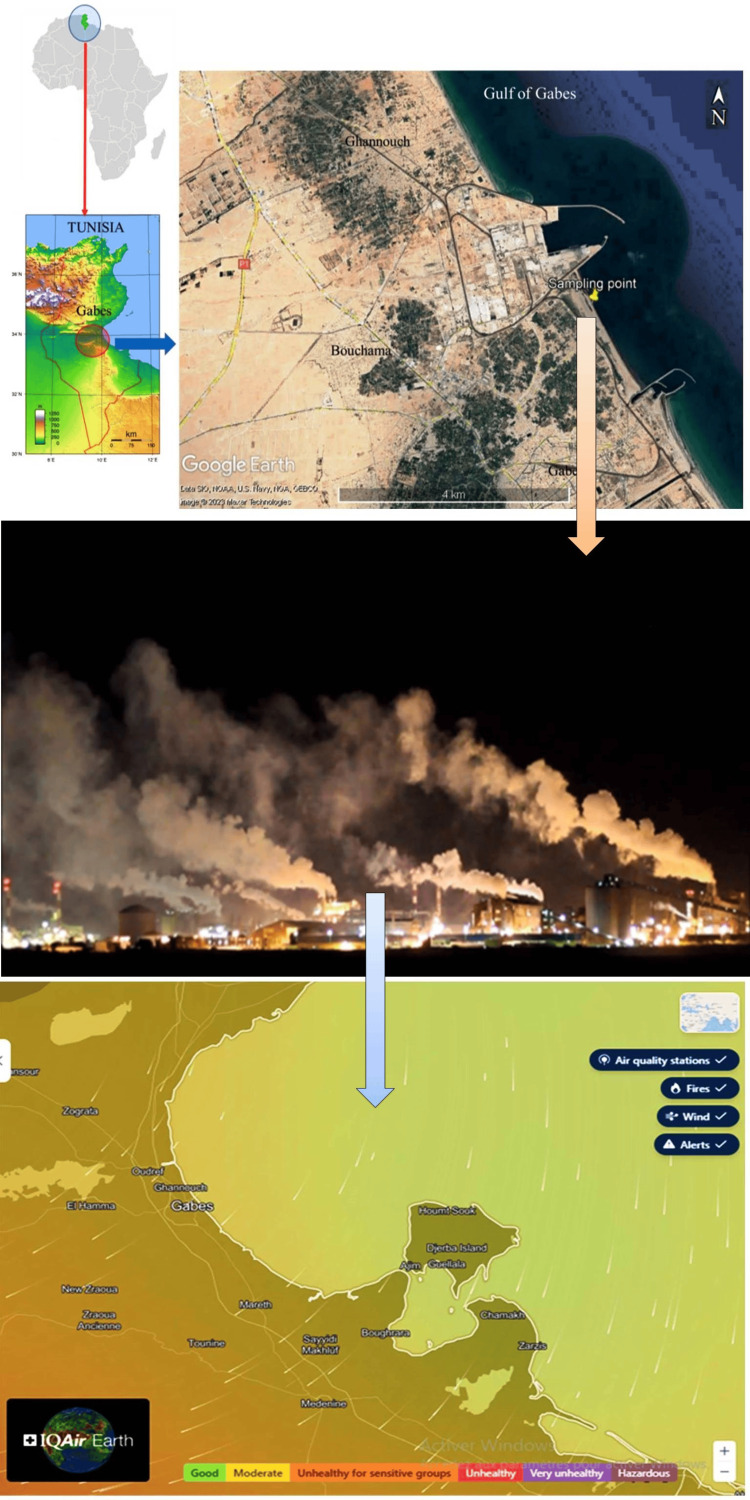
Study area of Gabès region Google Earth image [Google Earth NOAA GEBCO dataset (2023) for Ghannouch, Gabès (Southern Tunisia, *Longitude: 10° 04' 7.32" E, Latitude: 33° 52' 56.82" N, Altitude: 21 m*)] available at https://earth.google.com/web/ indicates the air quality sampling point for the study and Gabes, a city in South-East Tunisia, experiences high levels of air pollution due to organic and chemical pollutants from the industrial area in Ghannouch. The outdoor air quality monitoring station in Ghannouch provided daily concentrations of atmospheric pollutants for the study and the secondary climatic data from IQAir Earth (https://www.iqair.com/earth?nav) are also shown in Figure 1.

Daily concentrations of atmospheric pollutants, including particulate matter with an aerodynamic diameter ≤10 μm (PM10), sulfur dioxide (SO2), and ozone (O3), were collected from the Tunisian National Environmental Protection Agency. Additionally, daily data on moisture and ambient temperature were obtained from the same agency. The study was conducted in southeastern Tunisia, with the air monitoring station located in Gabès Ghannouch (Southern Tunisia, Longitude: 10° 04' 7.32" E, Latitude: 33° 52' 56.82" N, Altitude: 21 m), as shown in Figure 1. This region is characterized by significant levels of air and water pollution due to organic matter and chemical pollutants generated from the nearby industrial area [15-17].

Primary Outcomes and Severity Assessment

We extracted five primary outcomes: hospital admissions, severe pneumonia cases, the use of non-invasive ventilation (NIV), mechanical ventilation (MV), and pneumonia-related mortality. Severity was assessed using two measures: the CURB-65 severity assessment score, which includes criteria such as confusion (defined by an abbreviated Mental Test Score ≤ 8), blood urea nitrogen levels, respiratory rate, systolic and diastolic blood pressure, and age; and the Community-Acquired Pneumonia Severity Index (PSI). Outcomes also encompassed clinical and radiological evolution during hospitalization, the use of NIV and MV, and hospital mortality.

Statistical Analysis

The Statistical Package for the Social Sciences (SPSS) Version 20.0 (IBM Corp., Armonk, NY, USA) was used to perform the statistical analysis. The significance of correlations between datasets was determined using Pearson’s ‘r’ values. For each clinical and climatic measurement, a minimum of three replicates were conducted to ensure the reliability of the results.

## Results

The study involved 48 CAP cases presented to the Pulmonology Department of Gabès University Hospital. Table 1 summarizes the distribution of patients by month and season according to age. The average age of the patients was 63.79 ± 21.2 years, with a median of 69 years, and ranged from 18 to 92 years. Nineteen patients (40%) were aged 65 years or younger, while 60% (n = 29) were older than 65 years.

**Table 1:** Monthly patient admissions by age group

	Patient Age	
Admission Month	< 65 years (%)	> 65 years (%)
January	16	10
February	5	3
March	5	10
April	5	3
May	11	0
June	11	14
July	11	14
August	16	10
September	16	17
October	5	17

Our results indicated a significant seasonal variation in age distribution: the age group older than 65 years was predominantly represented in the autumn season, whereas the age group 65 years or younger was more prevalent in the summer. The gender distribution comprised 52% females and 48% males. Regarding lifestyle habits, 41% of the patients reported smoking. Additionally, 88% of the patients had a clinical history, with the most frequently reported antecedents being hypertension and diabetes mellitus.

Clinical presentation and outcomes

The average time from symptom onset to consultation was 5.17 days (range 1-15 days), with a median consultation time of 4 days (IQR 3-7 days). The average hospitalization duration was 6.08 days (range 1-30 days), with a median of 5 days (IQR 3-7 days).

The primary clinical presentations upon consultation were dyspnea (21 cases, 44%), cough (10 cases, 21%), fever (seven cases, 15%), chest pain (eight cases, 17%), and flu-like syndrome (two cases, 4%). Dyspnea was the predominant symptom, affecting 75% of the patients; it was progressive in 19 cases and acute in 17 cases. Cough was reported in 33 cases, with four cases presenting with dry cough. Cough with expectoration was noted in 28 cases, sputum was described as whitish in 15 cases, yellowish in eight cases, and greenish in five cases.

Table 2 illustrates the clinical findings upon admission, severity scores, and short-term complications of patients. Body temperature measurements revealed that 24 patients (50%) were febrile. Tachycardia was reported in 23 patients (48%), and tachypnea was noted in 30 patients (63%). Signs of respiratory distress were observed in 10 patients (21%). Abnormal pulmonary auscultation findings were noted in 43 patients (90%), with crackles being the most common. The mean Glasgow Coma Scale (GCS) score at admission was 14.75 ± 0.83, ranging from 10 to 15. One patient exhibited sensorimotor deficits, and five patients had GCS scores between 13 and 14. The average CURB-65 score was 1.17, and the average Pneumonia Severity Index (PSI) score was 85.6.

**Table 2:** Clinical examination findings, severity scores on admission, and acute complications in patients PSI: Pneumonia Severity Index

Clinical Examination findings on admission				
Parameter	Mean ± SD		Minimum	Maximum
Oxygen Saturation (SpO2)	90.36 ± 6.75		68	99
Heart rate (bpm)	99.11 ± 17.74		63	150
Systolic Blood Pressure (mmHg)	123.1 ± 22.1		70	190
Diastolic Blood Pressure (mmHg)	67.9 ± 13.7		40	110
Severity Scores				
Parameter	Mean ± SD		Minimum	Maximum
CRB65	1.17 ± 1.018		0	4
PSI	85.6 ± 37.418		10	199
Acute Complications				
Sepsis		2%		
Left Heart Failure		10%		
Hypertensive urgency		4%		
Acute Coronary Syndrome		2%		
Acute Renal Failure		13%		
Uncontrolled diabetes mellitus		29%		
Neurological deficits		4%		

Thirty-eight patients showed clinical improvement, with 19 successfully weaning off oxygen, a reduction in oxygen requirements observed in another 19, and resolution of fever noted in all cases. However, respiratory symptoms worsened in 10 patients, with persistent fever documented in six cases.

Among those with worsening respiratory symptoms, ventilator support was required for four patients - three required mechanical ventilation, and one patient was recommended for non-invasive ventilation. The immediate mortality rate was 4% (two cases). Radiological deterioration was observed in four cases, characterized by an extension of pulmonary opacity. Among these cases, two also presented with pleural effusion and one developed a lung abscess.

Climate data

**Figure 2:**
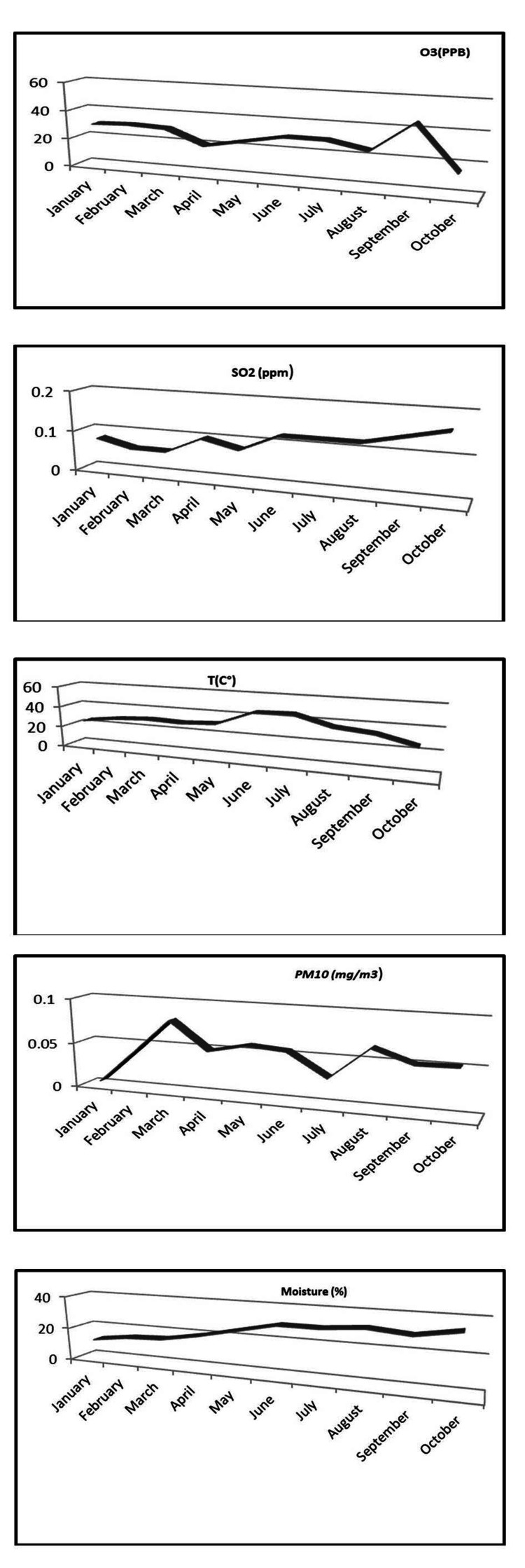
Climatic parameters Gabès region during January to October 2022 - PM10 (mg/m3), O3 (PPB), SO2 (ppm), temperature (C°) and air moisture (%)

Figure 2 illustrates the annual average climate parameters. The average PM10 level was 0.0544 ± 0.06 mg/m^3^, with a range from 0 to 0.98 mg/m^3^. The mean O3 value was 28.88 ± 0.4 PPB, with a range from 0.85 to 99 PPB. The average SO2 level was 0.109 ± 0.08 PPM, ranging from 0.0004 to 0.69 PPM. During the study period, the average temperature was 32.58 ± 8°C, and the average humidity rate was 17.55 ± 4%.

Correlation between clinical pneumonia and climatic parameters

A correlation analysis was conducted to investigate the impact of climatic changes on the aggravation of pneumonia, examining correlations between various clinical and climatic parameters depicted in Table 3.

**Table 3:** Correlation of climatic factors with clinical parameters SO2=Sulfur dioxide, O3=Ozone, PM10=Particulate matter diameter ≤10µm, BU=Blood urea, Creat=Serum creatinine, CURB65=Score for pneumonia severity, PSI=Pneumonia severity score, NIV=Non-invasive ventilation, MV=Mechanical ventilation *p<0.05, **p<0.01

Variable 1	Variable 2	R value
Asthenia	Dyspnea	-0.112
Asthenia	Chest X-ray abnormalities	-0.014
Asthenia	Lung Opacity	0.204
Asthenia	Pleural thickening	0.279
BU	Creat	-0.058
BU	CURB65	-0.025
BU	PSI	-0.004
Chest Pain	Asthenia	-0.272
Chest Pain	Dyspnea	-0.055
Chest Pain	Chest X-ray abnormalities	0.354*
Chest Pain	Lung Opacity	-0.322
Chest Pain	Pleural thickening	0.159
Creat	CURB65	0.194
Creat	PSI	0.322*
CURB65	PSI	0.824**
Dyspnea	Chest X-ray abnormalities	-0.06
Dyspnea	Lung Opacity	-0.165
Dyspnea	Pleural thickening	-0.283
Extensive damage on Chest X-ray or CT	Pleural effusion	0.148
Moisture	SO2	0.886**
Moisture	PM10	-0.215
Moisture	Temperature	0.602**
Moisture	O3	-0.033
Moisture	Chest Pain	0.014
Moisture	Asthenia	0.182
Moisture	Dyspnea	-0.36*
Moisture	Chest X-ray abnormalities	-0.07
Moisture	Lung Opacity	0.16
Moisture	Pleural thickening	0.286
Moisture	Radiological evidence of lung damage	0.007
Moisture	Extensive damage on Chest X-ray or CT	-0.298*
Moisture	Pleural effusion	0.382
Moisture	BU	-0.298*
Moisture	Creat	0.146
Moisture	CURB65	0.278
Moisture	PSI	0.31*
Moisture	Tachypnea	-0.117
Moisture	NIV	0.295
Moisture	VM	0.095
NIV	VM	-0.617**
O3	Chest Pain	-0.13
O3	Asthenia	0.094
O3	Dyspnea	-0.177
O3	Chest	-0.047
O3	Lung Opacity	0.036
O3	Pleural thickening	0.114
O3	SO2	0.049
O3	Moisture	-0.033
O3	Radiological evidence of lung damage	-0.07
O3	Extensive damage on Chest X-ray or CT	0.092
O3	Pleural effusion	-0.085
O3	SO2	0.049
O3	Moisture	-0.033
O3	BU	0.024
O3	Creat	0.124
O3	CURB65	-0.015
O3	PSI	-0.156
O3	SO2	0.049
O3	Moisture	-0.033
O3	Tachypnea	-0.196
O3	NIV	0.034
O3	VM	0.021
PM10	Temperature	-0.348*
PM10	O3	-0.125
PM10	Chest Pain	0.242
PM10	Asthenia	0.06
PM10	Dyspnea	0.151
PM10	Chest X-ray abnormalities	-0.054
PM10	Lung Opacity	-0.633**
PM10	Pleural thickening	-0.072
PM10	O3	-0.125
PM10	SO2	-0.329*
PM10	Moisture	-0.215
PM10	Radiological evidence of lung damage	-0.234
PM10	Extensive damage on Chest X-ray or CT	0.086
PM10	Pleural effusion	0.462*
PM10	O3	-0.125
PM10	SO2	-0.329*
PM10	Moisture	-0.215
PM10	BU	-0.002
PM10	Creat	0.088
PM10	CURB65	-0.088
PM10	PSI	0.025
PM10	O3	-0.125
PM10	SO2	-0.329*
PM10	Moisture	-0.215
PM10	Tachypnea	-0.008
PM10	NIV	-0.435*
PM10	VM	0.216
Radiological evidence of lung damage	Extensive damage on Chest X-ray or CT	0.442*
Radiological evidence of lung damage	Pleural effusion	0.187
SO2	PM10	-0.329*
SO2	Temperature	0.356*
SO2	O3	0.049
SO2	Chest Pain	0.043
SO2	Asthenia	0.142
SO2	Dyspnea	-0.291*
SO2	Chest X-ray abnormalities	-0.044
SO2	Lung Opacity	0.095
SO2	Pleural thickening	0.32*
SO2	Moisture	0.886**
SO2	Radiological evidence of lung damage	-0.024
SO2	Extensive damage on Chest X-ray or CT	-0.164
SO2	Pleural effusion	0.279
SO2	Moisture	0.886**
SO2	BU	-0.141
SO2	Creat	0.198
SO2	CURB65	0.299*
SO2	PSI	0.303*
SO2	Moisture	0.886**
SO2	Tachypnea	-0.143
SO2	NIV	0.4*
SO2	VM	0.087
Tachypnea	NIV	-0.717**
Tachypnea	VM	-0.617**
Tachypnea	Abscess	0.5
Temperature	O3	0.17
Temperature	Chest Pain	-0.192
Temperature	Asthenia	0.066
Temperature	Dyspnea	-0.458**
Temperature	Chest X-ray abnormalities	0.152
Temperature	Lung Opacity	0.706**
Temperature	Pleural thickening	0.094
Temperature	PM10	-0.348*
Temperature	O3	0.17
Temperature	SO2	0.356*
Temperature	Moisture	0.602**
Temperature	Radiological evidence of lung damage	0.169
Temperature	Extensive damage on Chest X-ray or CT	-0.19
Temperature	Pleural effusion	0.218
Temperature	PM10	-0.348*
Temperature	O3	0.17
Temperature	SO2	0.356*
Temperature	Moisture	0.602**
Temperature	BU	-0.272
Temperature	Creat	0.035
Temperature	CURB65	0.243
Temperature	PSI	0.241
Temperature	PM10	-0.348*
Temperature	O3	0.17
Temperature	SO2	0.356*
Temperature	Moisture	0.602**
Temperature	Tachypnea	0.131
Temperature	NIV	0.129
Temperature	VM	-0.16
VM	Abscess	0.5

The correlation study reveals numerous important associations that could help us understand environmental influences and pneumonia severity. A strong positive link between moisture and SO2 levels, as well as a significant correlation with temperature, implies that higher moisture and SO2 concentrations are connected with higher temperatures, potentially exacerbating pneumonia. An increase in SO2 levels was associated with a higher requirement for non-invasive ventilation (NIV) (r=0.400). Elevated SO2 levels were identified as a risk factor for severe pneumonia necessitating NIV. Increased SO2 levels were correlated with higher CURB-65 scores (r=0.299), signifying more severe pneumonia that requires hospitalization. Similarly, elevated SO2 levels were linked to higher Pneumonia Severity Index (PSI) scores (r=0.303), further indicating severe pneumonia necessitating hospitalization. Increased moisture levels were also associated with higher PSI scores (r=0.310).

Higher levels of PM10 were strongly correlated with the occurrence of lung abscesses, indicating that increased PM10 content is associated with a severe form of pneumonia. Additionally, elevated moisture and temperature levels were also significantly correlated with the development of lung abscesses. A positive correlation was found between PM10 levels and the occurrence of pleural effusion (r=0.462), suggesting that higher PM10 levels increase the risk of pleural effusion, which is associated with severe form of pneumonia. The substantial negative connection between PM10 levels and lung opacity suggests that higher particulate matter levels may be associated with decreased lung opacity, possibly indicating an interaction between air quality and lung inflammation or injury.

Furthermore, given the strong positive association between temperature and lung opacity, elevated temperatures may be associated with more severe lung involvement. The relationships between SO2 and other pneumonia severity measures, such as CURB65 and PSI, also imply that higher SO2 levels are associated with worse clinical outcomes. These findings underscore the complex interplay between environmental factors and pneumonia severity, suggesting that increased air pollution and temperature could intensify pneumonia symptoms and overall disease severity. Monitoring and mitigating environmental pollutants may be crucial for managing and preventing severe respiratory conditions.

## Discussion

Air pollution and long-term exposure to airborne contaminants are implicated in several respiratory system conditions. This study examined the association between outdoor air pollution exposure and the severity and outcomes of pneumonia requiring hospitalization. There was a higher number of older patients hospitalized during the autumn season. This finding aligns with previous studies, which attribute the age-related differences to immunological factors and greater exposure to risk factors among geriatric age [18].

Our results indicate a positive correlation between SO2 levels and the increased use of non-invasive ventilation (NIV), consistent with findings from similar studies [19, 20]. The associations between elevated SO2 levels and higher CURB-65 and Pneumonia Severity Index (PSI) scores, which indicate severe pneumonia requiring hospitalization, have also been corroborated by previous research [18].

Additionally, a positive correlation between PM10 levels and the occurrence of lung abscesses was observed, a relationship that has been reported in other studies [5]. The association between PM10 levels and pleural effusion was similarly supported by previous research [21].

Conversely, we found a negative correlation between climatic parameters (moisture and temperature) and the incidence of tachypnea (r = -0.360 and r = -0.448, respectively). This counterintuitive result may be explained by the hypothesis that increased moisture and temperature in our study region reduce air pollutant levels through thermodynamic processes in the atmosphere. Higher temperatures might denature or cleave atmospheric gases, thereby diminishing their harmful effects. Moreover, a negative correlation was found between chest X-ray opacity and PM10 levels (r = -0.633), suggesting that increased PM10 may reduce radiographic opacity, possibly due to particulate matter binding to and mitigating the effects of harmful gases. It is conceivable that the air quality in our study region affects PM10 as a risk factor.

Ozone did not show any significant correlation with clinical parameters in community-acquired pneumonia (CAP). This lack of correlation may be due to several factors. Firstly, the hot, arid climate of our study area could lead to the depletion of ozone levels, minimizing its potential impact as a risk factor. High temperatures in such regions are known to accelerate the breakdown of ozone in the atmosphere. Moreover, it is possible that acute, short-term spikes in ozone levels might not have the same effect as chronic, long-term exposure. The variability in individual susceptibility to ozone and the presence of other confounding factors, such as co-existing pollutants and pre-existing health conditions, might also obscure a clear correlation.

Limitations of the study

The study focuses on a single location (Gabes), which may limit the results' applicability to other places with varied environmental and industrial conditions. Other confounding variables, such as socioeconomic position, access to healthcare, pre-existing health issues, and lifestyle choices, may influence the severity and outcome of pneumonia but are not controlled for in the study. Changes in industrial activity or regulatory rules may also have an impact on pollution levels temporally.

## Conclusions

Addressing the relationship between acute community-acquired pneumonia severity and air pollution exposure requires proactive measures in air bioremediation to prevent further pollution and protect public health. It is imperative to advocate for renewable energy sources, enforce stringent industrial emission regulations, optimize transportation policies to reduce vehicle emissions, and establish comprehensive air quality monitoring systems. Equally important is enhancing public awareness of the health risks associated with air pollution and the critical need to reduce emissions. These integrated efforts aim to mitigate the adverse effects of air pollution on respiratory health and attenuate the severity of pneumonia and other respiratory illnesses.
